# Antioxidant Enrichment and Antimicrobial Protection of Fresh-Cut Fruits Using Their Own Byproducts: Looking for Integral Exploitation

**DOI:** 10.1111/j.1750-3841.2010.01792.x

**Published:** 2010-10

**Authors:** JF Ayala-Zavala, C Rosas-Domínguez, V Vega-Vega, GA González-Aguilar

**Affiliations:** Authors are with Centro de Investigación en Alimentación y Desarrollo, A.C. (CIAD, AC)Carretera a la Victoria Km 0.6, La Victoria. Hermosillo, Sonora (83000) Mexico. Direct inquiries to author Ayala-Zavala (E-mail: jayala@ciad.mx)

**Keywords:** antimicrobial, antioxidant, byproducts, fresh-cut fruits, safety and quality

## Abstract

Fresh-cut fruit consumption is increasing due to the rising public demand for convenience and awareness of fresh-cut fruit's health benefits. The entire tissue of fruits and vegetables is rich in bioactive compounds, such as phenolic compounds, carotenoids, and vitamins. The fresh-cut fruit industry deals with the perishable character of its products and the large percentage of byproducts, such as peels, seeds, and unused flesh that are generated by different steps of the industrial process. In most cases, the wasted byproducts can present similar or even higher contents of antioxidant and antimicrobial compounds than the final produce can. In this context, this hypothesis article finds that the antioxidant enrichment and antimicrobial protection of fresh-cut fruits, provided by the fruit's own byproducts, could be possible.

## Hypothesis Statement

Safety and the antioxidant value of fresh-cut fruits could be improved using the fruits’ own byproducts as a source of antimicrobial and antioxidant additives.

### Premise I: Fresh-cut fruits are an important source of antioxidants with a high risk of microbial spoilage

Recently, evidence that eating fresh fruits and vegetables is essential for good health and diet has been broadly shown in the literature (Hansen and others 2009). For example, a large number of epidemiological studies have demonstrated that people who eat a diet rich in fruits and vegetables have a lower risk of developing cancer (Steinmetz and Potter 1996; Hashimoto and others 2002), cardiovascular diseases (Vinson and others 1995) and chronic conditions (Sanchez-Moreno 2002), such as cataracts, asthma, and bronchitis (Theoharides and Bielory 2004). These beneficial effects have been attributed in part to the presence of bioactive compounds with antioxidant activity, such as phenolic compounds, carotenoids, and vitamins, which can delay or inhibit the oxidation of bio-molecules (DNA, proteins, and lipids).

Programs promoting the consumption of fruits that have been implemented by international public health offices, and the growing demand for easy-to-eat foods, have favored the increase in the sales of fresh-cut fruits (Hodge 2003). Another important factor that has influenced the demand for these products is the incorporation of most family members into the labor market; this has caused an increase in the number of meals that are eaten outside of the home. A convenient option for this is ready-to-eat food.

**Figure 1:**
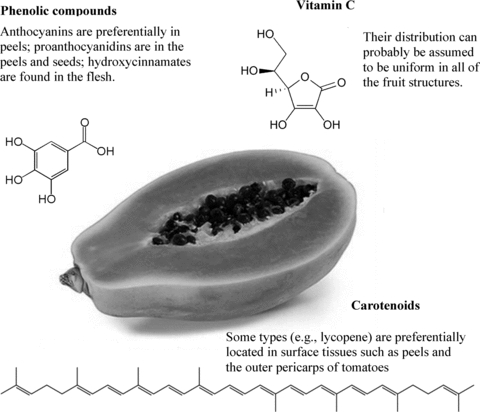
Major phytochemical compounds and their distribution in fruit tissues.

Currently, the most common fresh-cut fruit in the tropical regions is pineapple, melon, watermelon, apple, pear, and grape (Robles-Sánchez and others 2007). Besides their attractive colors, tastes, and aromas, tropical fruits have significant amounts of bioactive compounds with antioxidant capacity (Ajila and others 2010). Their general distribution in a fruit can be seen in Figure 1. However, the amount and concentration of individual bioactive compounds is a function of the type of cultivar, the maturity stage of the fruit, the storage conditions, preharvest handling, and their location among the different tissues in the same fruit.

The microbiological quality of fresh-cut fruits and vegetables is particularly critical given their exposure during the cutting process, which can cause contamination by bacteria, fungi, and yeast (Raybaudi-Massilia and others 2009). The commonly encountered micro-flora in fruits and vegetables is *Pseudomonas* spp., *Erwinia herbicola*, *Enterobacter agglomerans*, lactic acid bacteria, molds, and yeasts (Busta and others 2003). Among the deteriorative micro-flora, fungi are the most important microorganisms that cause the wasting of fresh-cut fruits because the relatively acidic conditions tend to suppress bacterial growth (Frazier and Westhoff 1993). On the other hand, most of the reported outbreaks have been associated with bacterial contamination, particularly by members of the family *Enterobacteriaceae*. The viruses involved in outbreaks have a human reservoir (for example, Norwalk-like and Hepatitis A) and can be associated with intact fresh products grown in contact with the soil and/or water (Busta and others 2003). Outbreaks linked to protozoa (for example, *Cryptosporidium, Cyclospora*, *Giardia*) have been more associated with fruits than with vegetables (Busta and others 2003). Protozoa and viruses are most often associated with contaminated water or food handlers, and they can be transmitted to the final fresh-cut produce during cultivation, harvest, cooling, storage, and minimal processing, which compromises consumer health (Garrett and others 2003). Thus, microbiological risk is one of the major factors affecting the quality and safety of fresh-cut fruits.

Food safety and quality have always been important to consumers and they continue to be a basic requirement of any modern food system. The chemical control of fresh-cut fruit decay (synthetic additives) has been used since the beginning of the food industry as a reliable preservative factor that controls the amount of deteriorative factors in fresh-cut fruits and vegetables. However, most of these compounds do not satisfy the concepts of “natural” and “healthy” that consumers prefer and that the food industry therefore needs to provide (Marriott 2010). This necessity is underlined by agro-industries, legislatures, and consumer organizations around the world.

### Premise II: Byproducts in the fresh-cut fruit and vegetable industry

The full utilization of horticultural produce is a requirement and a demand that needs to be met by countries wishing to implement low-waste technology in their agribusiness (Kroyer 1995). In the horticultural sector, there has been a growth in both acreage and agricultural production to fulfill the requirements of global food demand (Schieber and others 2001). This intensity of production generates large amounts of plant products, estimated to be around 800000 tons/y of fresh fruit and vegetable matter, without considering the wastage during processing. This might represent an important environmental problem if it is not addressed by the food industry (Ajila and others 2010). However, the integral exploitation of plant produce has not yet been achieved.

Vegetables and some fruits yield between 25% and 30% of nonedible products (Ajila and others 2007; Ajila and others 2010). The byproducts of fruits and vegetables are made up of skins and seeds of different shapes and sizes that normally have no further usage and are commonly wasted or discarded (Ajila and others 2007). In this context, the integral exploitation of the entire plant tissue could have economic benefits to producers and a beneficial impact on the environment, leading to a greater diversity of products directed to human usage (Schieber and others 2001). This situation can be extrapolated to different food processing areas, including the fresh-cut fruit industry.

Fresh-cut fruits and vegetables are products that must maintain a high percentage of their own attributes and quality parameters as compared to those of fresh whole products (IFPA 2002). These products are obtained by appropriate unit operations, such as washing, peeling, slicing, and packaging (Robles-Sánchez and others 2007). These production steps produce several byproducts that are normally wasted.

**Figure 2:**
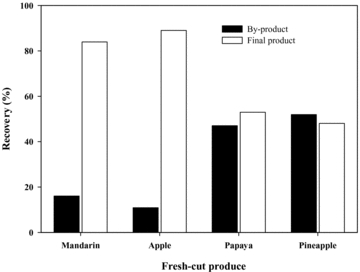
Percentage of recovery of fresh-cut fruits and byproducts.

Preliminary studies conducted in our lab demonstrated that several kinds of fresh-cut fruits produced variable amounts of byproducts to the extent even exceeding the quantity of end produce (Figure 2). The processed fruits were apples (*Malus domestica* cv. Golden Delicious), mandarins (*Citrus reticulata*), papayas (*Carica papaya* cv. Maradol), pineapples (Ananas comosus cv. Premium cayenne), and mangos (Mangifera indica cv. Kent). Sliced apples produced 10.91% of pulp and seed (core) byproducts and 89.09% of the final products. Peeled mandarins produced 16.05% of peels and 83.95% of final products. Diced papayas produced 6.51% of seeds, 8.47% of peels, 32.06% of unusable pulp (due to the lack of shape uniformity in a cube), and 52.96% of final products. Pineapples produced 9.12% of core, 13.48% of peels, 14.49% of pulp, 14.87% of top, and 48.04% of finished products. Mangos produced 13.5% of seeds, 11% of peels, 17.94% unusable pulp, and 57.56% of final products. It has to be highlighted that considerable amounts of fruit material are the byproducts of the minimal processing, and the possibility of creating alternative processes to give added value to this wasted material must be considered.

### Premise III: Antioxidant and antimicrobial potential of extracts derived from fresh-cut fruit and vegetable byproducts

**Figure 3:**
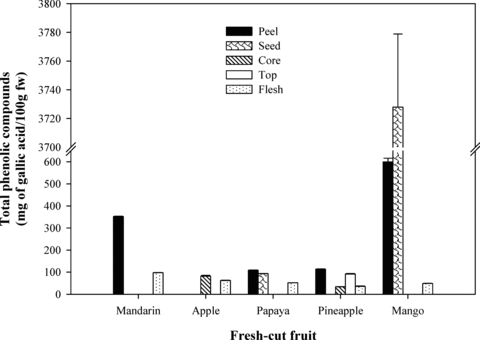
Total phenolic compounds of fresh-cut fruits and their byproducts. The concentrations of total phenolic compounds were measured by the methods described by Singleton and Rossi (1965). The concentration of total phenol compounds was calculated using a standard curve of gallic acid and expressed as milligram per 100 g of fresh weight.

**Figure 4:**
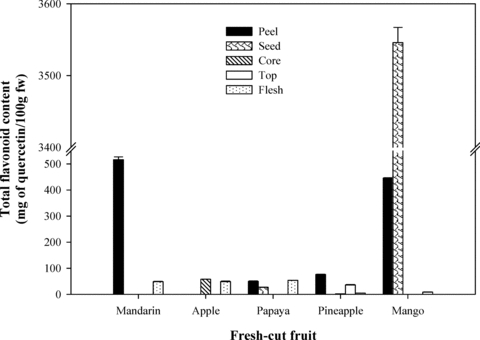
The total flavonoid content of fresh-cut fruits and their byproducts. The flavonoid content was determined based on the methods described by Zhishen and others (1999). The results were expressed on a fresh weight basis as milligram of quercetin equivalents per 100 g.

**Figure 5:**
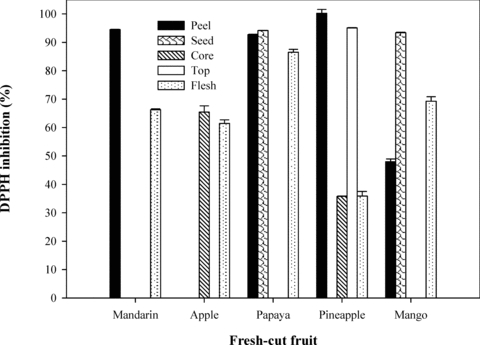
Radical scavenging activity (DPPH ^•^) of methanolic extracts of fresh-cut fruits and their byproducts (0.02 g/mL). The radical scavenging activity was expressed as the inhibition percentage of the DPPH ^•^ radical = (control OD – sample OD/control OD) × 100 (González-Aguilar and others 2007).

The most abundant byproducts of minimal processing of fresh-cut fruit and vegetable are peel and seed and those are reported to contain high amounts of phenolic compounds with antioxidant and antimicrobial properties (Shrikhande 2000; Gorinstein and others 2001; Muthuswamy and others 2008; Tuchila and others 2008). The products and byproducts obtained during the minimal processing of the fruits used in the preliminary studies mentioned earlier were analyzed for the phytochemical content and antioxidant status. Total phenolic and flavonoid content, and the stable radical inhibition DPPH were determined by the method of Singleton and Rossi (1965) (Figure 3), Zhishen and others (1999) (Figure 4), and González-Aguilar and others (2007) (Figure 5), respectively. It was found that the total phenolics and flavonoid contents were higher in the byproducts as compared with the final products, being more pronounced in mango seeds and peels. These compounds could be responsible for free radical inhibition activity, and those samples that showed the lowest contents of phenols and flavonoids also showed the lowest percentage of radical inhibition.

Several studies have shown that the content of phytochemical compounds is higher in peel and seeds with respect to the edible tissue (Table 1). The total phenolic compounds in the peels of lemons, oranges, and grapefruits were 15% higher than that of the pulp of these fruits (Gorinstein and others 2001). Eight selected clingstone peach cultivars were studied and it was reported that the peels contained 2 to 2.5 times the amount of total phenolic compounds as contained in the edible product (Chang and others 2000b). Peels from apples, peaches, pears as well as yellow and white flesh nectarines were found to contain twice the amount of total phenolic compounds as that contained in fruit pulp (Gorinstein and others 2001). While the edible pulp of bananas (*Musa paradisiaca*) contains 232 mg/100 g of dry weight phenolic compounds, this amount is about 25% of that present in the peel (Someya and others 2002). Similarly, other studies have reported that pomegranate peels contain 249.4 mg/g of phenolic compounds as compared to only 24.4 mg/g phenolic compounds found in the pulp of pomegranates (Li and others 2005). Apple peels were found to contain up to 3300 mg/100 g of dry weight of phenolic compounds (Wolfe and Liu 2003).

**Table 1:** Phenolic compounds found in different parts of fruits

Fruit	Part of the fruit	Phenolic compounds (mg/100 g)	Reference
Apple	Peel	3300.0*	(Wolfe and Liu 2003)
	Pulp	11800.0*	(Schieber and others 2003)
Avocado	Seed	8820.0*	(Soong and Barlow 2004)
	Pulp		
Banana	Peel	928.0*	(Someya and others 2002)
	Pulp	232.0*	
Clingstone Peach: cv. Andross	Peel	133.7**	(Chang and others 2000a)
	Pulp	41.5**	
Grape	Peel	5220.0**	(Bravo and others 1994)
Grapefruit	Peel	155.0**	(Gorinstein and others 2001)
	Pulp	135.0**	
Guava	Peel	5870.0**	(Jimenez-Escrig and others 2001)
Jackfruit	Seed	2770.0*	(Soong and Barlow 2004)
	Pulp	90.0*	
Lemon	Peel	190.0**	(Gorinstein and others 2001)
	Pulp	164.0**	
Longan	Seed	6260.0*	(Soong and Barlow 2004)
	Pulp	160.0*	
Mango	Seed	11700.0*	(Soong and Barlow 2004)
	Pulp	240.0*	
	Peel	7000.0**	(Larrauri and others 1996)
Orange	Peel	179.0**	(Gorinstein and others 2001)
	Pulp	154.0**	
Pomegranate	Peel	24990.0**	(Li and others 2005)
	Pulp	2440.0**	
Genotypes of tomato: 818 cherry^a^	Peel	40.0**	(George and others 2004)
	Pulp	27.0**	
DT-2	Peel	18.4**	
	Pulp	15.7**	
BR-124 cherry^a^	Peel	25.0**	
	Pulp	22.0**	
5656	Peel	26.7**	
	Pulp	23.0**	
7711	Peel	15.7**	
	Pulp	13.0**	
Rasmi	Peel	20.4**	
	Pulp	17.4**	
Pusa Gaurav	Peel	24.0**	
	Pulp	20.0**	
T56 cherry^a^	Peel	38.0**	
	Pulp	22.0**	
DTH-7	Peel	12.0**	
	Pulp	11.4**	
FA-180	Peel	12.7**	
	Pulp	11.7**	
FA-574	Peel	10.4**	
	Pulp	9.20**	
R-144	Peel	15.7**	
	Pulp	13.4**	
CD at 5%	Peel	2.86**	
	Pulp	1.33**	

a Cherry variety

* dry weight

** fresh weight.

Grape seeds and skins, the byproducts of grape juice and white wine production, are also sources of several phenolic compounds, particularly mono-, oligo-, and polymeric proanthocyanidins (Shrikhande 2000; Torres and Bobet 2001). It has been reported that the total phenolic compounds of seeds of several fruits, such as mangos, longans, avocados, and jackfruits, were higher than that of the edible product, and that the byproducts could be a valuable source of phytochemicals (Soong and Barlow 2004). The peels and seeds of tomatoes are richer sources of phenolic compounds than the pulp of the tomatoes are. The phenolic compounds of 12 genotypes of tomatoes has been studied, and, in general, lower levels were found in the flesh, ranging from 9.2 to 27.0 mg/100 g, as compared to 10.4 to 40.0 mg/100 g in the peels (George and others 2004). A similar observation was reported, and the total phenolic compounds (expressed as milligram of gallic acid equivalents per 100 g) of the skin, seeds, and pulp of tomatoes were found to be 29.1, 22.0, and 12.7 mg/100 g, respectively (Toor and Savage 2005). It was also found that the peel byproduct of tomato cultivars (Excell, Tradiro, and Flavorine) had significantly higher levels of total phenolic compounds, total flavonoids, lycopene, ascorbic acid, and antioxidant activity as compared with the pulp and seeds (Toor and Savage 2005). In general, it has to be highlighted that up to 10-fold higher occur between the phenolic contents of byproducts and the pulp.

The antimicrobial activities of a variety of naturally occurring phenolic compounds from different plant sources have been studied in detail (Burt 2004). These compounds play an important role in fruits’ protection against pathogenic agents, penetrating the cell membrane of microorganisms, causing lysis (Brul and Coote 1999; Ejechi and Akpomedaye 2005). Phenolic compounds from spices such as gingeron, zingerone, and capsaicin have been found to inhibit the germination of bacterial spores (Burt 2004). Polyphenols contained in green tea (*Camellia sinensis*) combat against *Vibrio cholerae* O1, *Streptococcus mutans, Shigella* (Si and others 2006). The antimicrobial activity of an ethanol extract from mango seed kernels against food-borne pathogenic bacteria has also been reported. The mango extract was more effective against gram-positive than gram-negative bacteria, with a few exceptions (Kabuki and others 2000). In addition, flavonoids have been reported to enhance the antibacterial, antiviral, or anticancer activities of compounds such as naringenin, acycloguanosine, and tamoxifen (Bracke and others 1999). The mixture of phytochemical constituents in plant extracts can be an advantage due to the synergistic effect that the constituents may have (Bakkali and others 2008).

Citric, succinic, malic, acetic, and tartaric acids are commonly found in fruits and fresh-cut byproducts. They have been traditionally used in the food industry as preservative agents, attributing their antimicrobial efficacy to the pH changes of the treated media (Raybaudi-Massilia and others 2009). In general, bacteria grow at a pH close to 6.5 to 7.5, but tolerate a pH range from 4 to 9 (Raybaudi-Massilia and others 2009). Yeasts are more tolerant to low pH values than bacteria are, whereas molds can grow in the widest pH range (Raybaudi-Massilia and others 2009). One effective way of limiting microbial growth is increasing the acidity of a particular food by adding an acidic substance (Raybaudi-Massilia and others 2009). Acids attack cell walls, cell membranes, metabolic enzymes, protein synthesis systems, and the genetic material of microorganisms (Tripathi and Dubey 2004).

### Premise IV: The application of bioactive extracts to the conservation of fresh-cut fruit

The usage of bioactive extracts as applied to fruit preservation is an alternative to chemical preservatives and helps to achieve consumer demand for fresh, nutritious and safe fruits, and vegetables that are free of synthetic additives. Presently, there are very few studies that provide information about the effect of bioactive compounds that are extracted from plant extracts and applied to fresh-cut fruits (Lanciotti and others 2004; Tripathi and Dubey 2004; Guillen and others 2007; Muthuswamy and Rupasinghe 2007; Martín-Diana and others 2008; Muthuswamy and others 2008; Raybaudi-Massilia and others 2009). However, the effect of antimicrobial and antioxidant extracts obtained from fresh-cut fruit byproducts as food preservatives has not been reported.

Some bioactive extracts have been proven to be effective antimicrobials and antioxidants; however, their addition to fruit may cause changes in sensorial attributes, as shown in Table 2. For example, green tea extract (GT) has been evaluated as being able to act in the preservative treatment of fresh-cut lettuce. Different quality markers, such as respiration, browning, ascorbic acid, and carotenoid content were evaluated. Several GT concentrations (0.25, 0.5, and 1 g/100 mL) at different temperatures (20 °C and 50 °C) were tested. Optimal GT treatments (0.25 g/100 mL at 20 °C) were compared with chlorine (120 ppm at 20 °C). High GT concentrations (0.5 g/100 mL and 1.0 g/100 m L) to a large extent prevented ascorbic acid and carotenoid losses of 0.25 g/100 mL GT as did chlorine. However, GT enhanced the browning of the samples, probably as a result of the high polyphenol content of the treatment, though heat-shock reduced this negative effect. No significant differences were observed between chlorine and the optimal GT (0.25 g/100 mL at 20 °C) in the browning appearance and sensory properties. GT kept the antioxidant activity of the samples better than chlorine did.

**Table 2:** Bioactive compounds and extracts applied to fresh-cut fruits and vegetables, considering sensorial, antimicrobial, and antioxidant effects

		Effects			
			
Bioactive compound	Fresh-cut fruit or vegetable	Sensory properties	Microbiological	Antioxidant	References
Ascorbic acid	Apple var. Gala	NM	Reduced up to 0.7 log CFU/g of *Salmonella ser.* Typhimurium, agona, and Michigan	NM	(DiPersio and others 2003)
Citrus oil	A mix of apple, pear, grape, peach, and kiwifruits	NM	Inhibited native microbiota and inoculated *Saccharomyces cerevisiae* by 17 d. Reduced the growth rate of *Escherichia coli*.	NM	(Lanciotti and others 2004)
Eugenol, thymol, and carvacrol	Grapes	Odor was detected after opening	Decrease of molds, yeasts and mesophilic aerobics	NM	(Rojas-Graü and others 2007)
Green tea extract	Lettuce	NM	NM	Prevents loss of ascorbic acid and carotenoids	(Martín-Diana and others 2008)
High fructose corn syrup containing calcium and zinc	Apple	Prevents browning discoloration. Increases in calcium and zinc	NM	NM	(Xie and Zhao 2003)
Lemon grass or oregano oils	Apple	NM	Reduction up to 4 log CFU/g of *Lysteria innocua*	NM	(Guillen and others 2007)

NM = nonmeasured effects.

In addition, in a study with grapes wrapped in 2 distinct films having different permeabilities, and treated with or without the addition of a mixture of eugenol, thymol, and carvacrol (Guillen and others 2007), the microbial counts (of molds, yeasts, and mesophilic aerobics) drastically decreased, and consequently diminished berry decay. Although a slight odor was detected after opening the packages, the typical flavors of those active compounds were not detected by trained panelists after tasting the berries. Thus, with this safe and simple technology, the overall quality (sensory and safety) of grapes could be improved significantly (Guillen and others 2007). Ethanol extract of cinnamon bark (1% to 2% w/v) and cinnamic aldehyde (2 mM) inhibited *Escherichia coli* O157:H7 and *Lysteria innocua in vitro* (Muthuswamy and others 2008). Ethanol extract of cinnamon bark (1% w/v) reduced the aerobic growth of bacteria inoculated fresh-cut apples significantly during storage at 6 °C up to 12 d. Catechin, chlorogenic acid, and phloridzin, 3 phenolic compounds that are abundant in apple processing byproducts, exhibited varying degree of inhibitory action toward the growth of tested food pathogenic and spoilage bacteria, fungi, and yeasts (Muthuswamy and Rupasinghe 2007). However, it is important to note that these phenolics (except 25 mM phloridzin) did not inhibit the probiotic bacterium *Lac. rhamnosus* suggesting no or minimal threat to the beneficial colon microflora, if the phenolics are used as food additives at the desirable concentrations. Also these authors suggest that the major phenolic compounds of apple byproducts could find use as food additives, however, the regulatory aspects of the use of plant extracts as fresh-cut fruit additives must be contemplated.

Regulatory actions are still being analyzed with respect to the use of natural plant extracts as food additives (Marriott 2010). Over the past decade, the demand for more natural food additives from consumers has resulted in an increase in the use of natural additives. This has now been reflected in changes to European legislation with the recent introduction of regulation EC/1334/2008. This regulation will be implemented in January 2011 and contained within this legislation are new definitions for natural extracts mainly with flavoring properties and processes that can be used in their preparation. In parallel to these changes there is increased scrutiny of traditional routes to extract preparations and a desire to move to cleaner and greener methods to extract natural compounds preparations. The U.S. Food and Drug Administration in the Code of Federal Regulations Title 21 refer to natural substances and natural adjuvants may be safely used in food in accordance with the following conditions. (a) They are used in the minimum quantity required to produce their intended physical or technical effect and in accordance with all the principles of good manufacturing practice. (b) In the appropriate forms (plant parts, fluid and solid extracts, concentrates, absolutes, oils, gums, balsams, resins, oleoresins, waxes, and distillates) they consist of one or more of the following, used alone or in combination with flavoring substances and adjuvants generally recognized as safe in food.

## Conclusion

The analyzed information showed that bioactive compounds from fresh-cut fruit byproducts could be used as natural additives to enrich antioxidant capacity while offering antimicrobial protection to the final fresh-cut produce. If this approach is realized, it would be possible to fulfill the requirements of the consumers of natural and preserved healthy and convenient fresh-cut fruits and vegetables, and the full utilization of the fruits could lead the industry to a lower-waste agribusiness, increasing industrial profitability through environmentally friendly operating processes.

To achieve this goal, future research and development efforts should address several objectives: Improve the international regulations on the use of plant extracts as food additives. Evaluate the economic feasibility of the alternative process of production of bioactive extracts from fresh-cut byproducts, contemplating the percentage and composition of the disposed material. Optimize and scale-up the extraction procedures of bioactive constituents, evaluate the effect of the extraction procedure (solvents, temperature, raw material) on the composition and activity of the obtained extracts, and identify the optimal application procedure and required doses to achieve both antimicrobial and antioxidant fortification without affecting sensorial acceptability. If sensorial acceptability of the treated fruit is affected, the use of odor–flavor masking technologies could be contemplated, like incorporation of extracts in edible coatings, encapsulation technologies, and controlled release systems. This new challenge must be considered by fruit processors, food technologists, and nutritional researchers in order to offer consumers new fresh products and assure the integral exploitation of fruit and vegetable material.
